# Reduction of Plasma Gelsolin Levels Correlates with Development of Multiple Organ Dysfunction Syndrome and Fatal Outcome in Burn Patients

**DOI:** 10.1371/journal.pone.0025748

**Published:** 2011-11-01

**Authors:** Li-feng Huang, Yong-ming Yao, Jin-feng Li, Ning Dong, Chen Liu, Yan Yu, Li-xin He, Zhi-yong Sheng

**Affiliations:** 1 Department of Microbiology and Immunology, Burns Institute, First Hospital Affiliated to the Chinese PLA General Hospital, Beijing, People's Republic of China; 2 Department of Obstertrics and Gynecology, Beijing Chaoyang Hospital Affiliated to Capital Medical University, Beijing, People's Republic of China; 3 11th Student Team, Undergraduate Medical School, Fourth Military University, Xi'an, Shanxi, People's Republic of China; Université de Technologie de Compiègne, France

## Abstract

**Background:**

Depletion of the circulating actin-binding protein, plasma gelsolin (pGSN) has been described in critically ill surgical patients. We hypothesized that the extent of pGSN reduction might correlate with different outcome of burn patients. The study was performed to evaluate the prognostic implications of pGSN levels on the development of multiple organ dysfunction syndrome (MODS) and fatal outcome in a group of severely burn patients.

**Methods and Findings:**

95 patients were included, and they were divided into three groups with different burn area: group I (n = 33), group II (n = 32) and group III (n = 30). According to whether there was development of MODS or not, patients were divided into MODS group (n = 28) and none-MODS group (n = 67); then the patients with MODS were further divided into non-survivor group (n = 17) and survivor group (n = 11). The peripheral blood samples were collected on postburn days (PBD) 1, 3, 7, 14, and 21. The levels of pGSN were determined and T cells were procured from the blood. The contents of cytokines (IL-2, IL-4 and IFN-γ) released by T cells were also measured. The related factors of prognosis were analyzed by using multivariate logistic regression analysis. The results showed that pGSN concentrations, as well as the levels of IL-2 and IFN-γ, decreased markedly on PBD 1–21, whereas, the levels of IL-4 increased markedly in all burn groups as compared with normal controls (P<0.05 or P<0.01), and there were obviously differences between group I and group III (P<0.05 or P<0.01). The similar results were found in MODS patients and the non-survivor group as compared with those without MODS and the survival group on days 3–21 postburn (P<0.05 or P<0.01). Moreover, as the pGSN levels decreased, the incidence of septic complication as well as MODS remarkably increased.

**Conclusions:**

pGSN levels appear to be an early prognostic marker in patients suffering from major burns.

## Introduction

Burn injury is one of the most common and devastating forms of trauma. Patients with severe burns require immediate specialized care in order to minimize morbidity and mortality. It has been demonstrated that a large thermal injury induces a state of immunosuppression that predisposes the patient to infectious complications [1]. It causes immune dysfunction involving both pro- and anti-inflammatory responses. The hallmark of the latter is a decrease in monocyte human leukocyte antigen-DR (HLA-DR) expression [2]. Meanwhile, enhanced microvascular permeability is also a fundamental component of the systemic inflammation that accompanies acute burn injury. Because actin is one of the most abundant proteins within cells (5–20% cell protein by weight for nonmuscle cells and 25% for muscle) [3], the amount of actin filaments released into the extracellular compartment during burn injury could conceivably be quite large. Previous studies showed that the infusion of actin monomers into rats caused pulmonary endothelial and vascular dysfunction [4]. Actin filaments could injure the microvasculature by either a direct effect on the vascular endothelium [4], [5] or by activating platelets [6] with resulting platelet aggregation, microvascular thrombosis, and perhaps the release of proinflammatory mediators such as thromboxane and lysophosphatidic acid (LPA).

Gelsolin, a protein of 82 to 84 kDa, is a member of gelsolin protein superfamily, which exists in a cytoplasmic as well as an excreted plasma isoform, and contains six homologous repeats termed gelsolin-like (G) domains [7]–[9]. Plasma gelsolin (pGSN) is the principal circulating protein which is able to sever and scavenge circulating filamentous actin [10]–[12]. In animal models, pGSN appears to be beneficial, possibly by virtue of its ability to counteract the pathophysiological consequences of actin release during trauma, shock and infection [13]–[16]. Further studies have revealed that critical extent of pGSN depletion in patients subjected to trauma, burns, major surgery or hematopoietic stem cell transplantation correlates with poor clinical outcome [17]–[19]. In addition, the finding that pGSN binds inflammatory mediators, including platelet activating factor as well as lysophosphatidic acid, suggests that its physiological function may be to localize inflammation and blunt its systemic effects, and that extensive pGSN depletion due to actin exposure following acute insults allows inflammatory mediators to cause widespread tissue damage [20]. Soon after traumatic injury, plasma concentrations of gelsolin are significantly reduced compared with those in healthy individuals [19]. Admission pGSN levels in patients with a variety of critical illness were associated with the development of multiple organ dysfunction syndrome (MODS) and septic shock [19], [21]. The main pathological conditions and the mechanisms which gelsolin involved in have been listed in Table 1.

**Table 1:** Gelsolin and diseases.

Related diseases	Main mechanisms linked to gelsolin
Acute inflammation	Gelsolin null mice respond more slowly to an induced inflammatory stimulus such as intraperitoneal thioglycollate instillation (Witke et al., 1995).The slower kinetics of leukocyte accumulation in gelsolin null mice were similar to those observed in P-selectin knockout mice (Mayadas et al., 1993) and most likely due to an impaired emigration of leukocytes from blood vessels (Witke et al., 1995). The blunted inflammatory response in gelsolin null mice is restricted to the early acute phase which suggests that gelsolin could represent a potential target for anti-inflammatory therapy (Witke et al., 1995).
Amyloidosis	One type of familial amyloidosis first identified in the Finnish population is caused by deposition of gelsolin (Maury et al., 2000; Maury et al., 2001). Plasma gelsolin isolated from homozygous Finnish type familial amyloidosis (FAF) patients lacks both, actin severing and nucleating activities (Weeds et al., 1993). In contrast, cytoplasmic gelsolin is not cleaved in these patients and the cellular actin modulating function of intracellular gelsolin is not affected (Kangas et al., 1999).
Chronic rheumatoid arthritis (RA)	Recent studies in the mouse as well as in patients have confirmed that gelsolin expression is lost in rheumatoid synovial fibroblasts leading to severe alterations in cytoskeletal organization (Aidinis et al., 2005). Furthermore, induction of RA in gelsolin null mice resulted in the exacerbation of the disease symptoms, suggesting that loss of gelsolin plays an important role in the pathophysiology of the disease.
Crohn's diseases	Smooth muscle from Crohn's patients change their phenotype from a contractile form to a migratory form that correlates with an increased expression of gelsolin (Ehrlich et al., 2000).
Cancer	Gelsolin expression is downregulated in 60–90% of tumors during carcinogenesis of breast, colon, stomach, bladder, prostate, and lung (Asch et al., 1999; Kuzumaki et al., 1997; Tanaka et al., 1995; Dosaka-Akita et al., 1998; Prasad et al., 1997; Sagawa et al., 2003). In these cancer types downregulation of gelsolin during tumor progression was observed and overexpression of gelsolin reverted the transformed phenotype in cell culture and mouse models (Sagawa et al., 2003). However, in a subset of breast tumors overexpressing the tyrosine kinase receptors erbB-2 and EGFR, gelsolin overexpression was described to correlate with negative prognoses (Thor et al., 2001).

Numerous studies show that an increased burn size leads to higher mortality in burn patients [22]. It was also implicated that the extent of burn size might be associated with the development of MODS [23]. The stress response to burn injury is similar to that of severe trauma or critical illness but differs in its severity and duration. The inflammatory response is triggered immediately after thermal injury and persists for almost 5 weeks postburn [24]. Superimposed severe infection can result in the disorder of one or more aspects of function of the host immune system and further induce suppression of immune function of T lymphocyte with shifting of Th1 (IL-2, IFN-γ) to Th2 (IL-4) following burn injury [22], [24]. However, little is known about the changes in pGSN level during the course of burn or its relationship with the extent of burns and the prognosis of the patient with severe burn. The aim of the study was to investigate the time course of pGSN levels in a relatively large sample involving 95 patients with severe burn and to determine whether the levels of pGSN could predict the immune states or outcome of patients.

## Methods

### Patient population and demography

95 patients who were consecutively admitted to our burn departments with total body surface area (TBSA) larger than 30% were included in the present study over a time period of 18 months. Patients were successfully resuscitated using colloid and lactated Ringer's solution. Within 96 hours of admission most patients underwent burn wound excision for full-thickness burn, and the excision wounds were seeded with granulated autologous skin, and totally covered with allograft. Five to ten days after healing of the donor area, the remaining wounds, if there was any, were totally covered with autograft skin.

The thermally injured patients were stratified into three groups according to burn size: 30%–49% TBSA burns (group I, n = 33), 50%–69% TBSA burns (group II, n = 32), and >70% TBSA burns (group III, n = 30). Demographic, laboratory, and clinical data were collected, and the Sequential Organ Failure Assessment (SOFA) score [25] was calculated, on admission and every 24 hours until postburn day (PBD) 21. In the calculation of the score, the worst values for each parameter in the 24-hour period were used. In sedated patients, the assumed Glasgow Coma Score Scale was used to evaluate the neurological status. The total SOFA was calculated as the sum of all daily SOFA scores for each patient. The mean score was defined as the ratio of total score to the length of stay (21 days) in the burn department. Failure of vital systems, such as respiratory system, circulatory system, renal and other systems, were recorded. Diagnosis of sepsis was confirmed by positive blood culture and the pathogen was identified during hospitalization or at autopsy, in combination with leucocytosis or leucopenia, hyperthermia or hypothermia, and tachycardia [26]. According to whether there was development of MODS or not, patients were divided into MODS group (n = 28) and non-MODS group (n = 67) based on the SOFA score and the criteria for definition of MODS [27]. Then the patients with MODS were further divided into non-survivor group (n = 17) and survivor group (n = 11). 25 healthy volunteers from the lab staff served as normal controls (17 men and 8 women, with mean age of 28.6±6.2 years, range 21–45 years). Whole blood samples (5 ml) were collected into EDTA-containing tubes from patients on PBD 1, 3, 7, 14, and 21 (patients died within PBD 21 were excluded from the study). After being centrifuged at 2,500 *g* for 5 minutes, plasma was harvested and frozen at −80°C until analysis. The study was reviewed and approved by the Institutional Review Board of the Burns Institute, First Hospital Affiliated to the Chinese PLA General Hospital, Beijing, China. Prior to the study, an informed consent was signed by the patient or next of kin. All patients and volunteers were of Chinese Han origin.

### pGSN measurements by ELISA

pGSN levels were measured using an enzyme-linked immunosorbent assay (ELISA), in accordance with the manufacturer's instructions (ELAab Science Co., Wuhan, China). The microtiter plate provided in this kit has been pre-coated with an antibody specific to GSN. Standards or samples were then added to the appropriate microtiter plate wells with a biotin-conjugated polyclonal antibody preparation specific for GSN. Next, avidin conjugated to horseradish peroxidase (HRP) was added to each microplate well and incubated. Then a TMB substrate solution was added to each well. Only those wells that contain GSN, biotin-conjugated antibody and enzyme-conjugated avidin would exhibit a change in color. The enzyme-substrate reaction was terminated by the addition of a sulphuric acid solution and the color change was measured spectrophotometrically at a wavelength of 450 nm. The concentration of GSN in the samples was then determined by comparing the O.D. of the samples to the standard curve. No significant cross-reactivity or interference was observed.

### Isolation of peripheral blood T lymphocytes

Heparinised blood was diluted in Hanks' balanced salt solution, and Ficoll-Hypaque (Sigma Chemical Co., St. Louis, MO) was used for isolation and preparation of peripheral blood lymphocytes. The isolated lymphocytes were washed twice with phosphate buffered saline (PBS), centrifuged (500 g, 4 minutes), resuspended in basal medium (unstimulated culture) RPMI-1640 (Gibco-BRL, Gaithersburg, MD) containing 10% fetal calf serum (Seromed, Berlin, Germany), 1% penicillin/streptomycin (Sigma Chemical Co., St. Louis, MO), and 1% glutamine (ICN, Eschwege, Germany). Then cell populations were counted under a light microscope. Based on the different experimental demand, the peripheral blood mononuclear cells (PBMC) suspension was diluted with RPMI-1640 to 2×10^6^ cells/ml. Cells were treated with 5 µg/ml Con A for 48 hours at 37°C in 5% CO2/100% humidified air, and the contents of cytokines [interleukin (IL)-2, IL-4 and interferon (IFN)-γ] released into supernatants were determined.

### Cytokine measurements by ELISA

IL-2, IL-4 and IFN-γ levels were determined by ELISA, strictly following the protocols provided by the manufacturer (Biosource, Worcester, MA). The color reaction was also terminated by adding 100 µl of ortho-phosphoric acid. Plates were read in a microplate reader (Spectra MR, Dynex, Richfield, MN).

### Statistical analysis

Data were expressed as mean ± standard deviation (SD) and analyzed with ANOVA (a mixed-model, factorial ANOVA) by means of SPSS 15.0 (SPSS Inc., Chicago, IL). Tukey Test was used to evaluate significant differences between groups. To assess the effect of pGSN levels and other independent variables on burn-derived complications and mortality, Logistic regressions were used as odds ratios (ORs) with 95% CI adjusting for the pGSN level, burn size, age, gender and inhalation injury. A *P* value of 0.05 or less was considered to indicate statistical significance.

## Results

### Demographics

95 patients with burn injury were included in the present study. The patients' demography was illustrated in Table 2. The test for homogeneity of variance was considered and the ANOVA assumption was met. The omnibus ANOVA was also found significant. There was no significant difference in age and gender among the patients with different burn sizes. However, there were significant differences in burn area between Group II and Group I (P<0.01). The MODS group had markedly larger burn area compared with the non-MODS group (P<0.01). Similarly, burn area in the non-survivors was much larger than that in the survivors (P<0.01). Both the length of hospital stay and the number of operations divided by the burn size were longest/largest in Group III, followed by Group II (P<0.01). Group III had significantly more patients with ventilator assistance requirements compared with the other two groups (P<0.01).

**Table 2:** Demographic and clinical characteristics of the patients.

Variable	Overall	Group I	Group II	Group III	MODS	Non-MODS	Survivors	Non-survivors
**n**	95	33	32	30	28	67	11	17
**Gender, n (%)**								
Male	69 (72.6)	21 (63.6)	26 (81.3)	22 (73.3)	19 (67.9)	50 (74.6)	8 (72.7)	12 (70.6)
Female	26 (27.4)	12 (36.4)	6 (18.7)	8 (26.7)	9 (32.1)	17 (25.4)	3 (27.3)	5 (29.4)
Male/Female	2.7	1.8	4.3	2.8	2.1	2.9	2.7	2.4
**Age (years)**	34.8±5.7	36.3±7.2	29.9±7.6	35.5±7.8	37.1±8.9	32.3±5.2	36.4±13.2	33.8±10.5
**Burn area (TBSA%)**								
Range	30–99	30–49	50–69	70–99	30–99	30–85	30–95	30–99
Mean area	55.3±8.2	37.5±6.8	58.2±12.9^**^	83.5±17.7^&&^	74.1±16.3^##^	47.5±9.7	55.6±19.7^††^	78.2±25.6
III° area	34.5±6.8	15.4±3.5	34.1±6.5^**^	61.3±14.9^&&^	53.3±16.4^##^	27.2±4.4	39.6±15.2^††^	59.2±17.1
**Length of stay (days)**	59.5±10.4	33.6±6.1	55.4±11.8^**^	72.5±15.1^&&^	65.6±18.5^##^	53.2±8.9	115.8±37.3^††^	31.2±12.7
**Operations (n)**	5.6±1.2	3.8±0.9	5.8±1.9^**^	8.2±2.4^&&^	6.9±2.6^##^	4.4±0.7	9.8±3.7^††^	5.7±1.9
**Inhalation injury,** **n (%)**	21(22.1)	3(9.1)	6(18.8)	12(40.0)	19(67.9)	2(3.0)	5(45.5)	14(82.4)
**Ventilator,** **n (%)**	17 (17.9)	1 (3.0)	4 (12.5)	12 (40.0)	17 (60.7)	0 (0)	8 (72.7)	9 (52.9)

The results were presented as the mean ± standard deviation or percentage. Tukey Test was used to evaluate significant differences between groups. TBSA, total body surface area; III° area, third-degree burn area; MODS, multiple organ dysfunction syndrome.

Significant difference between Group II and Group I (^**^P<0.01); Significant difference between Group II and Group III (^&&^P<0.01); Significant difference between MODS group versus Non- MODS group (^##^P<0.01); Significant difference between survivors group compared with non-survivors group (^††^P<0.01).

### Changes in pGSN levels after burns

**Figure 1:**
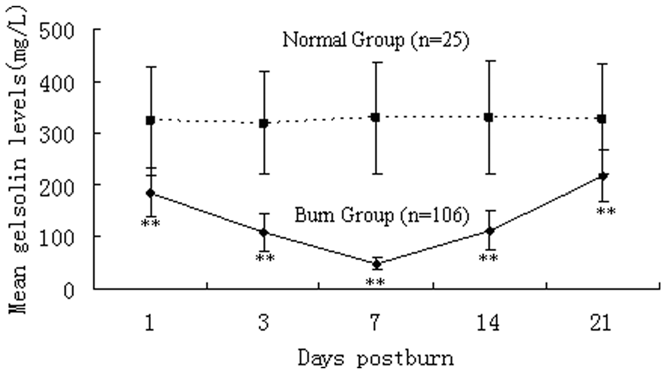
Time course of pGSN levels after burn injury. Time course of pGSN levels after burn injury. Mean pGSN levels were significantly decreased during PBD 1–21 compared with normal controls (all P<0.01), and there was a significantly lower level of pGSN on PBD 7 compared with other time points. **P<0.01, burn group vs. normal group.

**Figure 2:**
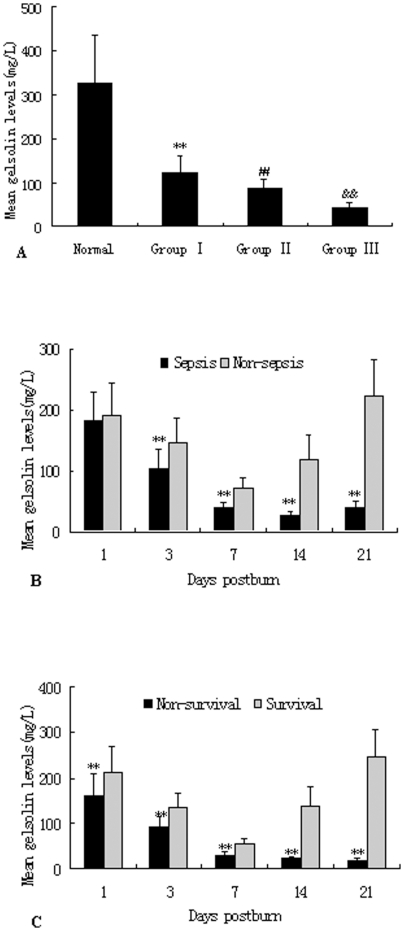
Comparison of pGSN levels in burned patients with or without MODS. Depressed pGSN levels (Fig. 2A) in burned patients were detected in comparison to normal controls, and there were obvious differences among patients with different extent of burn injury. pGSN levels (Fig. 2B) were much lower in patients with MODS than those without MODS on PBD 3–21. Among patients with MODS, pGSN levels in the survivors were obviously lower than those with non-survivors on PBD 1–21 (Fig. 2C). **P<0.01, Group I vs. normal group, or MODS group vs. non-MODS group, or non-survivors group vs. survivors group; ^##^P<0.01, Group II vs. Group I; ^††^P<0.01, Group III vs. Group II.

To investigate the changes in pGSN levels, the blood samples were detected by ELISA kits for human at different time points and in different groups after burns. As shown in Fig. 1, pGSN levels were significantly decreased in burned patients during PBD 1–21 compared with normal controls (P<0.01), and the lowest level of pGSN (45.28±7.22 mg/L) appeared on PBD 7. As shown in Fig. 2, there were significant differences in plasma levels of gelsolin among various groups. The pGSN levels were significantly lower in MODS patients than those without MODS during PBD 3–21 (P<0.01). Among patients with MODS, the pGSN levels in the survivor group were markedly higher than those with fatal outcome during PBD 1–21 (P<0.01). Among survivors the decreased pGSN levels appeared to partly retaliate after day 21 (244.51±51.95 mg/L), whereas pGSN levels remained low or even diminished further in the non-survivors with MODS (PBD 14: 19.46±4.98 mg/L and PBD 21: 15.61±4.17 mg/L).

**Figure 3:**
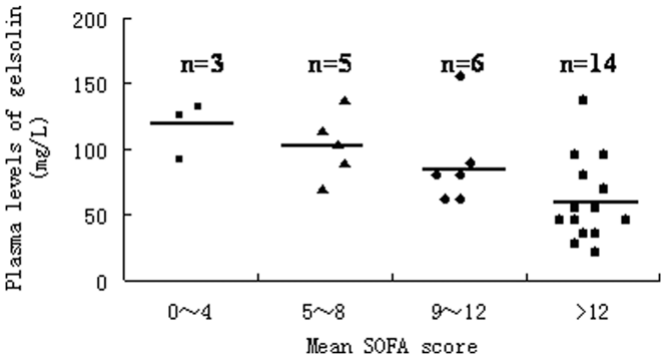
The relationship between pGSN levels and SOFA scores in patients with MODS. It was a comparision of the mean plasma gelsolin levels in 28 patients with different SOFA scores. The mean pGSN level in the >12 SOFA score group was 57.6±27.7 mg/L, which was significantly lower than those in other three groups (the 0–4 SOFA score group: 122.3±80.3 mg/L; the 5–8 SOFA score group: 101.5±52.7 mg/L; the 9–12 SOFA score group: 92.4±47.5 mg/L, all P<0.01).

The pGSN levels also gradually decreased with an increase in the mean SOFA score in MODS patients, as shown in Fig. 3. Meanwhile, as the pGSN levels decreased, the incidence of septic complication, as well as MODS, was remarkably increased. Patients with lowest pGSN levels (less than 100 mg/L) showed the highest rate of the development into MODS, and this correlation was more significant among patients who developed into sepsis and septic shock (Table 3).

**Table 3:** The relationship between pGSN levels and the incidences of sepsis and MODS in burned patients.

Variable	MODS				Sepsis (n = 55)	Septicshock(n = 16)
	Circulatory system failure(n = 21)	Respiratory system failure (n = 17)	Kidney failure (n = 11)	Failure of other systems (n = 35)		
**Gelsolin levels, n (%)**						
**Level I**	11(52.4)	8(47.1)	5(45.5)	18(51.4)	29(52.7)	14(87.5)
**Level II**	7(33.3)	6(35.3)	5(45.5)	9(25.7)	19(34.5)	2(12.5)
**Level III**	3(14.3)	3(17.6)	1(9.0)	8(22.9)	7(12.8)	0(0)

Mean pGSN levels during PBD 1–21 were determined in MODS and septic patients.

Level I: < or = 100 mg/L, Level II: 100–200 mg/L, Level III: > or = 200 mg/L.

### Changes in protein levels of cytokines released by T cells

**Figure 4:**
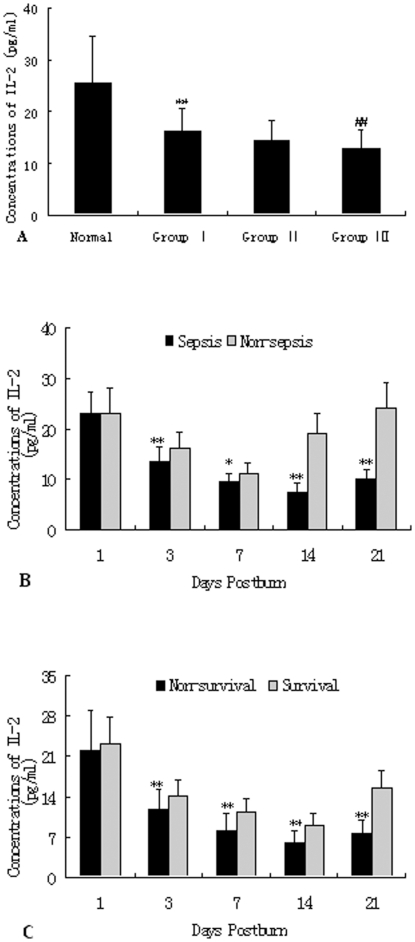
ELISA analysis of IL-2 levels released by T cells. Decreased expression of IL-2 released by T cells from burned patients was noted on PBD 1–21 in comparison to normal controls, and there were obvious differences among patients in Group I and Group III (Fig. 3A). Protein levels of IL-2 of T cells were significantly lower in patients with MODS than those without MODS on PBD 3–21 (Fig. 3B). Among patients with MODS, IL-2 levels in the survivors were obviously higher than those with non-survivors on PBD 3–21 (Fig. 3C). *P<0.05, **P<0.01, Group I vs. normal group, or MODS group vs. non-MODS group, or non-survivors group vs. survivors group; ^##^P<0.01, Group III vs. Group I.

**Figure 5:**
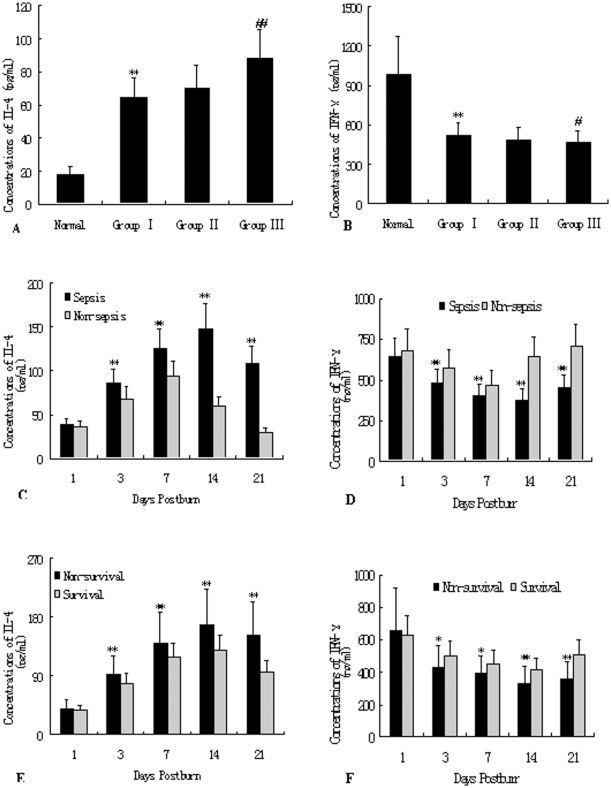
ELISA analysis of of IL-4 and IFN-γ released by T cells. Levels of IL-4 produced by T cells response to PHA increased markedly after burn injury, whereas levels of IFN-γ decreased markedly as compared with normal control group, and there were obvious differences between group I and group III, indicating that Th cells might have shifted to Th2 cells (as shown in Fig. 4A and 4B). The similar results were obtained in non-MODS patients and the survival group as compared with those with MODS and the non-survival group on PBD 3–21 (as shown in Fig. 4C∼4F). *P<0.01, **P<0.01, Group I vs. normal group, or MODS group vs. non-MODS group, or non-survivors group vs. survivors group; ^#^P<0.01, ^##^P<0.01, Group III vs. Group I.

As shown in Fig. 4, decreased expression of IL-2 released by T cells from burned patients was detected on PBD 1–21 in comparison to normal controls (25.63±8.95 pg/ml, P<0.01), and there were significant differences among patients in Group I and Group III (17.36±5.28 pg/ml versus 11.92±3.44 pg/ml; P<0.01). Protein levels of IL-2 released by T cells were significantly lower in patients with MODS than those without MODS on PBD 3–21 (P<0.05 or P<0.01). Among patients with MODS, IL-2 levels in the survivors were significantly higher than those with non-survivors on PBD 3–21 (P<0.01). As shown in Fig. 5A and 5B, levels of IL-4 produced by T cells in response to phytohemagglutinin (PHA) increased markedly after burn injury, whereas levels of IFN-γ decreased markedly as compared with normal control group(IL-4: 17.64±5.20 pg/ml, IFN-γ: 984.40±292.52 pg/ml, all P<0.05), and there were obvious differences between group I and group III (IL-4: 62.49±18.22 pg/ml versus 91.54±18.29 pg/ml, P<0.01; IFN-γ: 522.77±132.28 pg/ml versus 452.30±96.71 pg/ml, P<0.05), indicating that Th cells might have shifted to Th2 cells. As shown in Fig. 5C–5F, the similar results were found in non-MODS patients and the survival group as compared with those with MODS and the non-MODS group on PBD 3–21 (P<0.05 or P<0.01).

### Independent predictors for the development of MODS or fatal outcome

The related factors of prognosis were analyzed by using multivariate logistic regression analysis. The results showed that the pGSN level, burn size, age and inhalation injury were independent predictors for the development of both MODS and fatal outcome of burn patients (P<0. 05, Table 4). Gender showed no independent association with MODS and mortality of patients (P>0. 05, Table 4).

**Table 4:** Multiple logistic regression models of independent risk factors for MODS and fatal outcome of burn patients.

Variable	MODS		Fatal outcome	
	Odds ratio (95%CI)	*P* value	Odds ratio (95%CI)	*P* value
**pGSN level**	5. 7 (2. 7∼12. 3)	0.000	5.5 (2. 4∼12.0)	0.000
**Burn size**	4.7 (2. 0∼11. 0)	0.000	4.1(1. 8∼9.4)	0.001
**Age**	4.9 (1. 8∼13.4)	0.002	3.8 (1.6∼9. 0)	0.003
**Gender**	2.3 (1. 1∼5.9)	0.087	3.0 (1. 6∼6. 9)	0.179
**Inhalation injury**	2. 7 (1. 2∼6. 0)	0.021	3.7 (1. 3∼9. 9)	0.008

## Discussion

Severe burn injury may induce a temporal shift in immune reactivity that can lead to septic syndrome or even death. The immune system responds to injury by a rapid production of early and late proinflammatory cytokines, and also suppression of T cell functions [28]. Despite significant advances in intensive care technologies and high enthusiasm in the development of new antibiotics, severe sepsis and MODS after major burns still claims 40%–50% mortality in many countries [23], [29]. Therefore, it is of great significance to further elucidate the pathogenetic mechanisms, and to seek novel interventional strategies to prevent and treat sepsis or MODS secondary to severe trauma or burns.

The actin cytoskeleton is an essential scaffold for integrating membrane and intracellular functions. It is very dynamic and is remodeled in response to a variety of signals. Gelsolin was first identified as a cytoplasmic actin-regulatory protein essential for cell locomotion and phagocytosis. When a circulating isoform was subsequently discovered, its physiological significance was far from clear. For over two decades, researchers have made concerted effort to define the *raison d'être* for actin-binding proteins in plasma, which under normal circumstances does not contain detectable actin. A straightforward hypothesis emerged suggesting that pGSN scavenges actin which leaked into the circulation or interstitial space from injured tissue in order to abort subsequent damage instigated by extracellular actin filaments [30]. In the study conducted by Lee and coworkers [16], depletion of pGSN in animal models of sepsis occurred within 6 hours after a septic challenge with either endotoxin (lipopolysaccharide) or a multiple microbes challenge after cecal-ligation and puncture [16]. Several clinical studies [17], [21] have observed that a low gelsolin level after an initial insult, such as injury or inflammation, reflected greater severity of disease and poorer outcome. The depletion of pGSN soon after a septic challenge may result from exposure of the actin cytoskeleton, which occurs as part of cellular injury [31]–[33]. In turn, depletion of gelsolin could allow the formation of actin filaments, which would lead to further tissue injury and multiple organ dysfunction [4], [30], [34]–[38].

Accordingly, the degree of pGSN depletion might reflect the extent of tissue injury leading to significant exposure of actin to the extracellular space after major burns. In the current study, a decreased level of pGSN from burned patients was also observed on PBD 1–21 compared with normal controls, and there were obvious differences among patients with different burn sizes. This finding was consistent with the result of a previous report in an animal model, showing a significant reduction in circulating gelsolin levels within the first 2 hours of injury, with the decrease persisting for at least 6 days postburn in burned rats [39]. The diminution in pGSN levels after acute burn injury may reflect the leakage of gelsolin, as well as other plasma proteins, across the microvasculature at the site of the burn and in other microvascular beds [40], and it enhanced clearance of gelsolin-actin complexes by the liver [12]. The higher rates of loss of pGSN relative to total plasma proteins at the later time points suggest that pGSN is selectively removed from the circulation, that it is not recycled once it leaked out of the vasculature, or that it is not replenished by *de novo* synthesis. As we know, pGSN is synthesized primarily in skeletal muscle [11], and burn injury promotes skeletal muscle degradation [41]. It is unlikely that the reduction in circulating gelsolin levels is due to expansion of the intravascular fluid compartment by the infusion of crystalloid resuscitation fluids. This notion is supported by the observations that pGSN concentrations remained significantly reduced even when normalized to circulating plasma protein levels, and the hematocrit of burn injured patients was never less than that of the control group, as one would expect had hemodilution been present. Irrespective of how circulating gelsolin is decreased during burn injury, the absolute reduction in the amount of pGSN would limit its protective effects against inflammation for a prolonged period.

Based on our findings, it was indicated that a burn injury involving larger than 50% TBSA caused remarkably prolonged length of stay in hospital, significant increase in number of operations, and incidence of infection and MODS. Indeed, several investigators also found that low pGSN levels were associated with poor outcome in trauma [19] and critically ill surgical patients [18], [42]. The present study focused on the clinical implications of pGSN levels obtained after admission. We demonstrated that, although almost all the patients had decreased gelsolin levels after extensive burn injuries, the lowest levels were associated with less SOFA score and more complicated disease courses (e.g. sepsis or septic shock). We alao found that the pGSN levels were lower in patients with MODS than those without MODS and healthy control individuals. Moreover, as the pGSN levels decreased, the incidences of septic complication as well as MODS remarkably increased. It seemed that the pGSN level might correlate with occurrence of severe sepsis and MODS, and it did significantly differ between surviving and nonsurviving patients with MODS. In the current study, recovery of pGSN levels was observed late in the course in survivors but not in non-survivors with MODS, thus gelsolin levels might be a discriminative index in this context. On one hand, the gelsolin level provides an earlier or more reliable marker of the development of MODS than do conventional parameters; on the other hand, there are biologically plausible reasons to implicate gelsolin as a potential physiological mediator in the evolution of poor outcome in burn patients.

Severe thermal injury induces detrimental changes in immune function, often rendering the host highly susceptible to development of life-threatening opportunistic infections. Multiple mechanisms have been proposed to explain infection-induced immunosuppression, including an imbalance in the cellular helper T cell (Th1/Th2) or cytokine profile, induction of anergy, depletion of effector cells and most recently the activation of CD4^+^CD25^+^ Tregs [43]. In our previous experiments [44]–[46], significant proliferation of splenic T cells and IL-2 as well as IL-2Rα expression on T cells were simultaneously suppressed to certain extent after burns. Current dogma has expanded to now accommodate the convincing observations that pGSN can bind a variety of potentially inflammatory moieties, and can thereby modulate the exuberance of the host response to sepsis, malaria, burns, trauma, and other commonly encountered clinical conditions [34], [36], [39], [42]. It is well established that major burn injury *per se* can trigger both excessive inflammation and suppressed adaptive immunity. Actin exposed at the site of an overwhelming injury may act as a sink to exhaust the supply of pGSN, in turn allowing redundant inflammatory cascades unfettered opportunity to wreak havoc upon multiple organ systems. After a phase of excessive immune activation, a marked apoptosis-induced depletion of lymphocytes and a nonspecific anergy of immune function subsequent to severe insults might be responsible for the increased susceptibility of the host to subsequent septic complications. Similarly, our results in this study showed that the levels of IL-4 produced by T cells increased markedly after burn injury, whereas levels of IL-2 as well as IFN-γ decreased markedly, indicating that Th cells might have shifted to Th2 cells.

These observations of 95 patients with burn injuries of different extent confirmed the strong correlation between pGSN levels and such important clinical complications as the development of MODS and fatal outcome. Still unsettled issues concern the particular or incremental utility of gelsolin levels as timely prognostic indicators, the generalization of these findings to diverse clinical situations [21], and the biological consequences resulting from hypogelsolinemia. Rothenbach et al [13] found that intravenous infusion of recombinant human gelsolin completely prevented an increase in pulmonary microvascular permeability in burn injured rats. This study provides additional evidence to support the notion that circulating gelsolin has an important preventive effect on the pathophysiology of systemic inflammatory states after severe burns. The potential therapeutic benefits of infusions of recombinant human pGSN for patients in whom sepsis and multiorgan dysfunction commonly follows a serious insult have not yet been investigated. Nevertheless, it was evident that gelsolin repletion to physiological levels between the time of the initial insult and subsequent complications in patients identified by marked hypogelsolinemia as being at substantial risk for delayed multiorgan dysfunction could provide a directed and effective therapeutic intervention in critically ill patients.

### Conclusions

In conclusion, pGSN may be a valuable marker for severe complications in patients suffering from major burns. Extensive burn injury could result in significantly decreased pGSN levels, which appears to be associated with the development of MODS and fatal outcome secondary to acute insults. It also suggested that the potential therapeutic benefits of infusions of recombinant human pGSN might be important to the Th1/Th2 cytokine balance in patients after extensive burns. The prognostic role of pGSN in a critical illness requires further investigation in a large cohort.
